# Supplementary material for: Splice-Junction-Based Mapping of Alternative Isoforms in the Human Proteome
Source: Cell Rep. Author manuscript; Available in PMC 2020 Jan 15. (PMC6961840; doi:10.1016/j.celrep.2019.11.026)
Supplement: 2 [file NIHMS1546469-supplement-2.zip › DF1/PXD009737/TestisTrypsin/TestisTrypsin_42_MTX3_LSLGDGWGGGWGLPSVHSESLVVMAYAK.pdf]

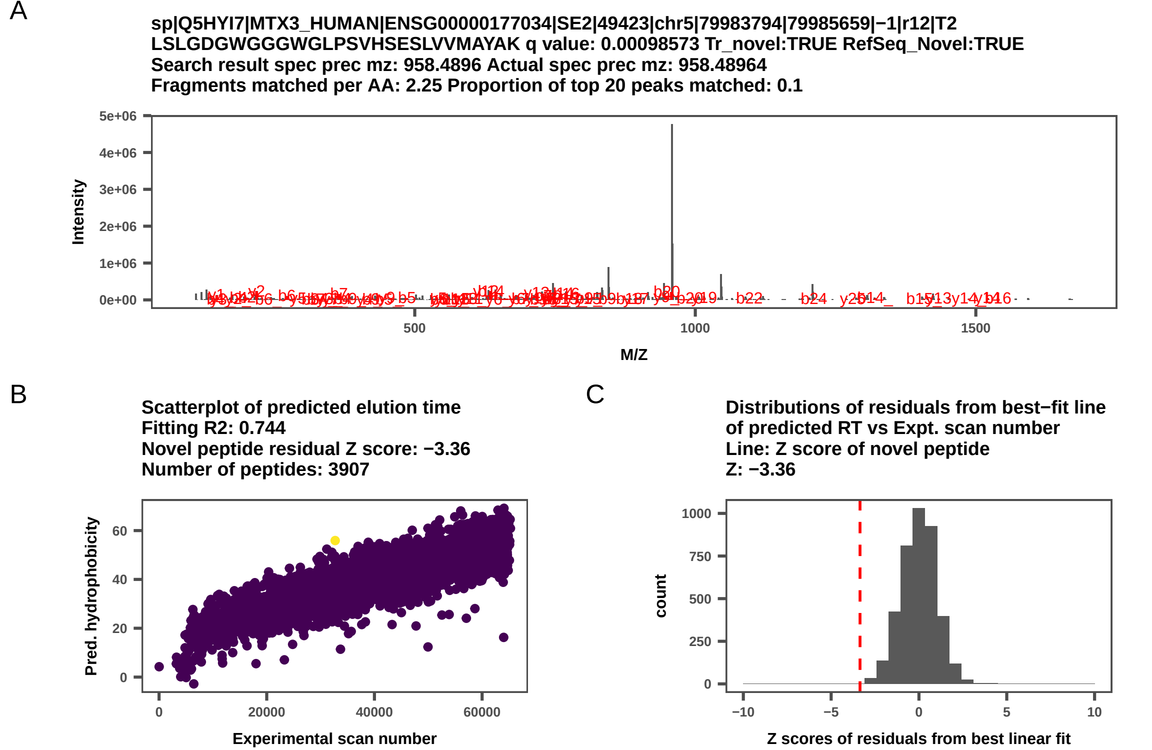
A
sp|Q5HYI7|MTX3_HUMAN|ENSG00000177034|SE2|49423|chr5|79983794|79985659|−1|r12|T2
LSLGDGWGGGWGLPSVHSESLVVMAYAK q value: 0.00098573 Tr_novel:TRUE RefSeq_Novel:TRUE
Search result spec prec mz: 958.4896 Actual spec prec mz: 958.48964
Fragments matched per AA: 2.25 Proportion of top 20 peaks matched: 0.1
Intensity
5e+06
4e+06
3e+06
2e+06
1e+06
0e+00
500
1000
1500
M/Z
B
Scatterplot of predicted elution time
Fitting R2: 0.744
Novel peptide residual Z score: −3.36
Number of peptides: 3907
Pred. hydrophobicity
60
40
20
0
0
20000
40000
60000
Experimental scan number
C
Distributions of residuals from best−fit line
of predicted RT vs Expt. scan number
Line: Z score of novel peptide
Z: −3.36
count
1000
750
500
250
0
−10
−5
0
5
10
Z scores of residuals from best linear fit
